# Comparing Life Satisfaction of Immigrants and Natives Across Europe: The Role of Social Contacts

**DOI:** 10.1007/s11205-017-1629-x

**Published:** 2017-04-20

**Authors:** Bruno Arpino, Helga de Valk

**Affiliations:** 1 Department of Political and Social Sciences and The Research and Expertise Centre for Survey Methodology (RECSM), Universitat Pompeu Fabra, Carrer Ramon Trias Fargas 25-27, 08005 Barcelona, Spain; 2 NIDI - The Netherlands Interdisciplinary Demographic Institute/KNAW/University of Groningen, The Hague, The Netherlands

**Keywords:** Life satisfaction, Social embeddedness, Immigrants, Second generation, Europe, European Social Survey

## Abstract

Research on immigrants’ assimilation is widespread both in the U.S. and Europe. While it has been extensively studied how immigrants fare compared to natives on socio-economic indicators, few studies have focussed on immigrants’ perception of their position. In this paper we focus on comparing life satisfaction of immigrants and natives across Europe and on the role of social embeddedness. Using data from the first six rounds (2002–2012) of the European Social Survey, a repeated cross-sectional survey, we find that life satisfaction among immigrants is lower than among natives even though differences diminish over generations. For first generation immigrants part of the life satisfaction gap is explained by the lower level of social embeddedness they have compared to natives. We also find that social embeddedness is a key explanatory factor for life satisfaction for both immigrants and natives. For two out of the three indicators of social embeddedness that we consider we however find different patterns of association with life satisfaction for immigrants compared to natives.

## Introduction

Research on immigrants' assimilation is widespread both in the United States and Europe. While it has been extensively studied how immigrants behave compared to natives in terms of employment, earnings, educational attainment and other objective socio-economic indicators (for an overview for Europe see e.g., Van Tubergen et al. 2004; Heath et al. 2008), few studies have focussed on immigrants’ perception of their status and their subjective wellbeing. This is especially the case for Europe where migration, health and wellbeing so far have been rather understudied (Rechel et al. 2011). Few past studies have addressed the question of whether immigrants are satisfied with their life as compared to natives in the society of residence (Simpson 2011). This is unfortunate as growing shares of European populations are of immigrant origin. Not including them in studies on life satisfaction and wellbeing does not do justice to the growing ethnic diversity in many European countries (de Valk et al. 2012; Van Mol and de Valk 2016).

In this study we focus on immigrants´ life satisfaction across Europe and compare immigrants of different generations to those of the majority population in the country. Human perception is fundamental to the definition of wellbeing and it can be argued that the only person who really knows whether a person is feeling well is the person itself (Layard 2005). Better health, quality of work and relationships, freedom of choice and political participation, a higher degree of trust in one’s community are all reported to contribute to higher life satisfaction. Social relations and contacts are furthermore found to be key for life satisfaction among majority groups (Kahneman and Riis 2005).

How social relations are relevant for life satisfaction among immigrants and their descendants is still only partially understood. Many studies based on socio-economic indicators have shown that immigrants are behaving more similar to natives over time (e.g., Heath et al. 2008). However, the latter seems to be less the case for norms and values (Lesthaeghe and Surkyn 2006). Also the scarce existing studies on life satisfaction suggest this is not happening. Safi (2010) showed for Europe that immigrants report significantly lower levels of life satisfaction than natives and this gap does not disappear by immigrant generation or length of stay (similar findings are reported by Baltatescu 2005; Kirmanoglu and Baslevent 2014). She partially explained this by discrimination experienced by immigrants in the country of destination. Her findings therewith point to the importance of relationships and social embeddedness of migrants for their subjective wellbeing as also previous studies among majority groups have indicated (Kahneman and Riis 2005). No previous study to our knowledge has taken this dimension into account for immigrant populations. However, social relations might be even more crucial for understanding life satisfaction among this group given their migration experience and the related changes in social relationships due to the move (Nauck 2000). Life satisfaction is a relevant aspect of immigrants’ life as it may also show how they perceive their live and as such it may therefore be a better proxy of immigrants’ conditions or at least it can integrate traditional objective indicators of adaptation.

In this paper we focus on life satisfaction of immigrants and natives across European countries by taking a comparative approach. We introduce three innovative aspects that have been largely overlooked in studies so far. First, we assess to what extent immigrants (from diverse generations) have different levels of satisfaction with their lives than the native majority group in the country of residence. Second, we aim to explain differences between immigrants and natives by looking at the social capital and embeddedness of the person. Previous work has reported the important role of social networks for immigrants’ economic performance (Kanas et al. 2012) as well as for their choices in the family domain (Huschek et al. 2011). Third, we will consider whether social capital plays a different role for life satisfaction across immigrant generations and natives. In fact, gaps in life satisfaction levels between immigrants and natives may be in part explained because for immigrants the protective factors for loneliness of social relations in family and social support are less strong (Koelet et al. 2016). Analyses of how subjective wellbeing differs by (immigrant) origin and levels of social relations is not only crucial for expanding theory in these domains but may just as well provide indicators for social policies in which quality of life and well-being have become key issues nowadays in Europe. The data for the analyses come from the European Social Survey. The pooled first six waves (2002–2012) of data collection allow for comparing life satisfaction of migrants and non-migrants across Europe as well as the differentiating the factors associated with it including social capital, the main focus of our work.

## Life Satisfaction: Concepts and Findings

Studies on life satisfaction and wellbeing are numerous and all refer to the individuals’ evaluation of his or her life. Being satisfied with one’s life can help to manage stress and add to well functioning in society. Often a distinction is made between on the one hand emotional well-being or affect (the emotional quality of an individual’s everyday experience, the frequency and intensity of experiences of joy, stress, sadness, anger and affection that make one’s life pleasant or unpleasant) and on the other hand life evaluation or cognition (the thoughts that people have about their life when they think about it) (e.g., Fleurbaey et al. 2009; Kahneman and Deaton 2010). Previous experiences in life can be important for the evaluation of one’s life. Studies have distinguished between the potential effects of experiences versus stability of happiness over time within individuals as a characteristic of the person. Studies have in the meantime shown we should study both together: Wellbeing is both a personality trait as well as a balance of happy and unhappy experiences the individual has over life (Ormel et al. 1999).

Life satisfaction is a broader indicator of a person’s wellbeing at some point, it is a “cognitive based judgement of life” (Ormel et al. 1999, p. 76). Different measures for life satisfaction have been developed. The Satisfaction with Life Scale (SWLS) for example assesses satisfaction with the respondent’s life as a whole. The scale does not assess satisfaction with life domains such as health or finances separately but allows subjects to integrate and weigh these domains the way they choose. It assesses an individuals’ conscious evaluative judgment of his or her life by using the person’s own criteria (Pavot and Diener 1993). The SWLS has been shown to be psychometrically sound and it shows good convergent validity with other scales and with other types of assessments of subjective well-being (see e.g., Diener 1984 or Pavot and Diener 2008 for a review). Also Larsen et al. (1983) found that single life satisfaction measures did not seem to be influenced substantially by social desirability. The scales do however correlate with personality measures, with happiness ratings made about respondents by others and with other non-self-report data (Weinstein 1982).

## Life Satisfaction Among Migrants

The limited existing research on life satisfaction among immigrant groups has in particular concentrated on understanding increases in levels of happiness after migration as a consequence of realised expectations of better economic and social living standards (Bartram 2013). At the same time existing studies on migrants after they have moved show in general lower levels of life satisfaction among them compared to the majority group (Baltatescu 2005; Safi 2010; Kirmanoglu and Baslevent 2014). The reasons for lower levels of life satisfaction are found in both migration specific factors as well as contextual host society effects. Recent studies suggest that life satisfaction also differs between first and second generation migrants (Safi 2010; Kirmanoglu and Baslevent 2014) as well between those who are less or more integrated (the latter being happier with life). So far most studies on immigrants have mainly focused on one national context (see Safi 2010 and Kirmanoglu and Baslevent 2014 for exceptions) like Germany (Obućina 2013; Angelini et al. 2014) or Israel (Amit 2010) or one particular immigrant group in terms of origin (see Baltatescu 2007).

Although studies have acknowledged regional variation in happiness (Bjørnskov 2008), countries are found to be more homogeneous with regard to general life satisfaction (Cummins 1995; Plaut et al. 2002). This suggests that life satisfaction is at least partly dependent on cultural mechanisms that are present in society and can be understood as part of a cultural process of interpretation and ways of giving meaning to life. At the same time this implies that the level of individualism versus collectivism orientation in a society (see e.g., Hofstede 2001) may be important for the measured life satisfaction. In more individualistic societies wellbeing is perceived to be an individual responsibility and may therefore result in a positive bias towards reporting higher levels of subjective well-being and life satisfaction (Diener et al. 1995; Inglehart 1997).

In line with this type of explanations one could expect that cultural assimilation of immigrants coming from more collectivistic countries migrating to more individualistic societies may influence their life satisfaction. Indeed a study in Germany found that the life satisfaction gap between Germans and immigrants is due to different levels of cultural assimilation and identification with Germany among immigrants (Angelini et al. 2014): Those who feel more integrated and identify more with Germans are more satisfied with life than those who do not. However, this effect was only found for those who resided in Germany for a longer period of time or who belonged to the second generation. Similar findings were also reported for the Netherlands with respect to differences between immigrant origins (Gokdemir and Dumludag 2012). The difference between the subjective wellbeing among Turkish versus Moroccan immigrants in the Netherlands (with the latter reporting higher levels of wellbeing) was found to be attributable to their levels of identification with the Netherlands.

Studies are however inconclusive about the duration of stay and generational effects on life satisfaction. Several studies have found a positive correlation between life satisfaction of migrants and their *duration of stay* abroad (Erlinghagen 2011; Bartram 2013) whereas others find that the second generation is less satisfied with life than the first generation (Safi 2010). Continued symbolic boundaries between ethnic groups in societies across Europe are found to have an important effect on happiness according to a recent study by Beier and Kroneberg (2013) but they only are relevant for those migrants who are having language problems in the host society. Suggesting again that integration and links to the host society are crucial for life satisfaction over and above cultural difference, period of stay and generation (Simpson 2011).

Indeed previous studies also indicated that host society affects life satisfaction of immigrants. Psychologists too have acknowledged that evaluations of the individual self (e.g., in terms of intelligence and happiness) depends on the context of comparison (see Mussweiler 2003 for a review; Diener and Diener 2009 for the importance of context to well-being). A study based on the SOEP data in Germany showed that life satisfaction decreased when right wing extremism among the native population increased (Knabe et al. 2013). Also the cross-national comparative studies by Safi (2010) and Kirmanoglu and Baslevent (2014) reported, based on ESS data, that higher levels of discrimination are related to lower levels of life satisfaction.

### The Role of Social Capital

Social wellbeing is according to Ormel et al. (1999) dependent on status control, confirmation of behavior and affection. In this paper we focus in particular on this last aspect for which social relations play a key role. Social support has been identified essential in people’s social wellbeing (Siedlecki et al. 2014). Life satisfaction in this view depends on the congruence of achievements and aspirations. This raises particularly interesting questions in an immigration context as migrants may choose their fellow migrants, the native population or the situation in their country/region of origin as the counterfactual against which to measure their own lives. The links they have with different social groups and networks they are part of can in this way be relevant.

Social capital in social science refers to the impact of networks on society and individuals (Putnam 1993, 2000). In migration sociology there is a longstanding interest in the role of social networks in migration processes (e.g., Thomas and Znaniecki 1918; Petersen 1958; Völker et al. 2008; van Tubergen 2014). Studies have focused on the impact of social networks on migration decisions (e.g., Boyd 1989; Massey and Espinosa 1997) as well as on the role of social networks after migration with settling in the host society for example for finding a job (e.g., Cook et al. 2011) and housing (e.g., Gill and Bialski 2011). However no attention so far has been paid to the importance of social capital and social networks for life satisfaction among migrants and non-migrants.

This is quite surprising as social relations and social capital may foster a sense of rootedness and integration (Korinek et al. 2005). Many authors have pointed to the positive effects of social embeddedness for the individual and the position in society (e.g., Coleman 1988; Granovetter 1992; Portes and Sensenbrenner 1993; Langford et al. 1997; Korinek et al. 2005). Socially embedded individuals can rely on group solidarity and informal social support for the pursuit of their personal goals. Migration can on the one hand have a disruptive effect on these social relations (Coleman 1990). On the other hand it can also strengthen social ties as migrants’ existing ties do not necessarily disappear but will be maintained (Levitt 2001). In a migration context, however, contact with the majority population is often considered to be crucial for the social integration of immigrants in society (Völker, Pinkster and Flap 2008). Although research on the relation between social capital and subjective well-being is quite scarce, it is well established that inclusion in society and social support are essential for well-being (Helliwell and Putnam 2004; Winkelmann 2009).

Putnam (2000) differentiated between ‘bonding’ and ‘bridging’ social capital and pointed to the importance of bridging ties when studying immigrants’ integration. Ties with the local population in migrants’ social network would serve as a bridge to integration and would result in a better position in the host society (de Miguel Luken and Tranmer 2010). Establishing social relations after migration however takes time and is potentially difficult (Putnam 2000; Lubbers et al. 2010; Nisic and Petermann 2013). Nevertheless if these ties are not established migrants are prone to loneliness, lower perceived quality of life, lower levels of life satisfaction, lower levels of happiness, negative perceptions of self and others, anxiety, and depression (Galent et al. 2009; Vancluysen and Van Craen 2011). Levels of social integration, identification and migration reasons were indeed found to be key in explaining differences in life satisfaction of migrants of different origins in Israel (Amit 2010, 2012).

It can thus be expected that social capital will positively influence subjective well-being (Helliwell and Putnam 2004; Hooghe and Vanhoutte 2011). In a study on Belgium (focusing only on the majority group) Hooghe and Vanhoutte (2011) report indeed positive effects of social relations on life satisfaction. Having social capital contributes to life satisfaction over and beyond individual and socio-demographic characteristics as well as personality (in terms of a more positive view on life) according to their analyses.

When studying life satisfaction among migrants one of the key issues is the identification of the correct reference group in the analyses (Simpson 2011; Lessard-Phillips et al. 2015). Here we are first of all interested in comparing migrants with the majority group in the society they live in as a way to assess the effect of migration on life satisfaction. Second we assess life satisfaction of immigrants across generations to identify changes with the group of immigrants as studies emphasize the role of adaptation for a range of individual outcomes (Heath et al. 2008; Safi 2010). Finally, we examine whether and how different levels of social embeddedness explain the gap and the potentially different effects of social capital for individuals of the majority group and those of immigrant origin.

## Data and Methods

We use data from the European Social Survey^1^ (ESS), a unique comparative survey that allows for comparisons both between migrants and non-migrants and across immigrant generations. The ESS is a repeated cross-sectional survey implemented every second year since 2002. We pooled the data of the six waves covering 2002–2012 for the purpose of our analyses. The ESS has been developed aiming for a fully comparative European perspective and great effort has been made in the translation of questionnaires to ensure comparability across the participating countries. In total 34 European countries have participated in at least one wave of the survey.

The ESS is representative of the population aged 15 or older. We restricted our sample to people aged 18–65, which results in an initial sample of 224,263 individuals. We excluded 19,316 individuals (8.6%) with missing information on one or more variables used in the statistical analyses leading to a final sample of 204,947 respondents.

Our dependent variable is a generalized measure of life satisfaction which is asked in all waves of the ESS allowing for full comparability. Although we acknowledge the multi-facettedness of life satisfaction and the fact that multiple indicators may yield more reliable results (Kahneman and Krueger 2006) more detailed information on different aspects of life satisfaction is not available for (all waves of) the ESS and we therefore focus on this more limited measure. Nevertheless also Hooghe and Vanhoutte (2011) note that this more restricted measure captures quite some of the subjective well-being of the person.

Life satisfaction was measured by using a standard question: *All things considered, how satisfied are you with your life as a whole nowadays?* In the ESS, this variable is measured with a 11-point Likert scale ranging from 0 (extremely dissatisfied) to 10 (extremely satisfied).

We classify immigrants based on the country of birth of the individual and both parents resulting in three groups: first generation (G1, immigrants who were born outside of the current country of residence); second generation (G2, children of immigrants who have both foreign born parents); 2.5 generation (G2.5, children of immigrants with only one foreign born parent). Natives are the reference category in the regression analyses.

Starting from the second round of the ESS, the country of birth of both the respondents’ father and mother is asked if one of the two was born in a country different from the country of the survey (in addition to the country of birth of first generation immigrants, G1). However, in the first wave only the continent of origin of foreign-born parents was asked. Therefore, to avoid losing information from wave 1 we refrained from covering countries of origin but do include in our multivariate analyses a categorical variable indicating the continent of origin: Africa, Asia, Europe (reference), North America, South America, Oceania.

Social embeddedness, that is the main focus of this paper, was measured by including the following three questions from the survey: *how often do you meet socially with friends, relatives or work colleagues?* (Never = 1, Less than once a month = 2, Once a month = 3, Several times a month = 4, Once a week = 5, Several times a week = 6, Every day = 7)*; Compared to other people of your age, how often would you say you take part in social activities?* (Much less than most = 1, Less than most = 2, About the same = 3, More than most = 4, Much more than most = 5); *Do you have anyone with whom you can discuss intimate and personal matters?* (no = 0, yes = 1).

We also controlled for additional covariates that were shown to be explanatory factors of life satisfaction in previous studies (see Hooghe and Vanhoutte 2011 for an overview of the literature). These indicators were shown to be important for life satisfaction among the majority population and we thus include them in our analyses as controls.

We assume that these factors have a similar effect for migrants as they have for the majority group. First, partnership status (in a partnership—the reference category, never married, separated or divorced, widowed), and the presence of children in the home (no = 0, yes = 1) are included. We also controlled for gender (men being the reference group) and age using a set of dummy variables: 18–29, 30–54 (reference), 55–65. Respondents’ education level was measured using the highest level of education completed, using the International Standard Classification for Education (ISCED) and specified as dummy variables in the analyses: less than secondary, lower secondary, upper secondary or some post-secondary (reference), and tertiary education. We also controlled for activity status (employed—reference, in school, unemployed, retired, other) and the type of area of residence (big city, small city, rural—reference) by including these as dummy variables in the analyses.

In a first step we carried out descriptive analyses. This is followed by multivariate analyses, based on linear regression models (OLS) with wave, country of residence and continent fixed effects. In this way we control for institutional, cultural and economic contextual factors that may influence life satisfaction.

## Results

### Descriptive Analyses

Table 1 reports the descriptive statistics for all independent variables by immigrant generation. In our sample, immigrants tend to be younger than natives. In particular, the percentage of people in the oldest category (55–65) is lower among immigrants. Immigrants are also more concentrated in big cities and show a higher unemployment rate as compared to natives. As for the independent variables that are of key interest here, we find slightly lower levels of social embeddeddness for first generation immigrants as compared to the other groups on all three indicators (differences are significant at the 1% level). At the same time we do not find systematic differences between natives and the second generations (G2 and G2.5).

**Table 1:** Descriptive statistics (%) of the covariates by immigrant generation (N = 204,947)

Covariates	Immigrant generation			
	G1	G2	G2.5	Natives
Meet (M)	4.9	5.2	5.1	4.9
Activities (M)	2.6	2.7	2.7	2.7
Intimate discussion	90.5	92.0	92.8	92.4
Female	45.2	46.3	46.7	46.9
Age				
18–29	19.5	26.6	26.0	22.2
30–55	62.4	57.6	55.9	56.9
55–65	18.1	15.7	18.1	20.9
Type of area of residence				
Big city	45.6	52.5	38.9	31.8
Small city	31.6	29.0	31.5	30.1
Rural	22.9	18.5	29.6	38.1
Education level				
Less than secondary	9.0	4.5	4.2	8.8
Lower secondary	16.0	14.9	13.4	16.2
Upper secondary	40.2	51.5	50.1	48.1
Tertiary	34.8	29.0	32.3	27.0
Activity status				
Employed	61.0	61.4	61.4	62.0
In school	6.3	10.2	9.5	7.2
Unemployed	9.6	8.2	7.2	7.2
Retired	6.9	6.7	8.1	9.2
Other	16.2	13.6	13.8	14.4
Partnership status				
With partner	59.7	54.0	48.4	55.8
Never married	24.9	32.4	35.6	30.7
Separated	12.1	11.1	12.8	10.0
Widowed	3.3	2.4	3.2	3.6
Child in home	52.0	51.0	45.2	46.6
Continent of origin				
Africa	11.8	17.3	6.4	
Asia	16.7	20.3	8.1	
Europe	60.4	57.1	76.9	
Norh America	2.1	0.8	2.7	
South America	6.3	2.5	1.9	
Oceania	0.6	0.1	0.4	
N	18,503	5763	10,574	170,107

For categorical covariates we report the percentage corresponding to each category

For numerical variables we report the mean (M)

The majority of immigrants in our sample have a European origin (around 60% of G1 and G2 and 77% of G2.5; See Table 1). On average, 17% of each country sample is composed of immigrants (ranging from 2.6% in Bulgaria to 50% in Luxembourg) and the share of immigrants in our sample is fairly stable across waves (from 15.7% in wave 2 to 18.3% in wave 5; data not shown). We will adjust for these factors by including dummy variables for the continent of origin, country of residence and wave in the regression models.

**Fig. 1:**
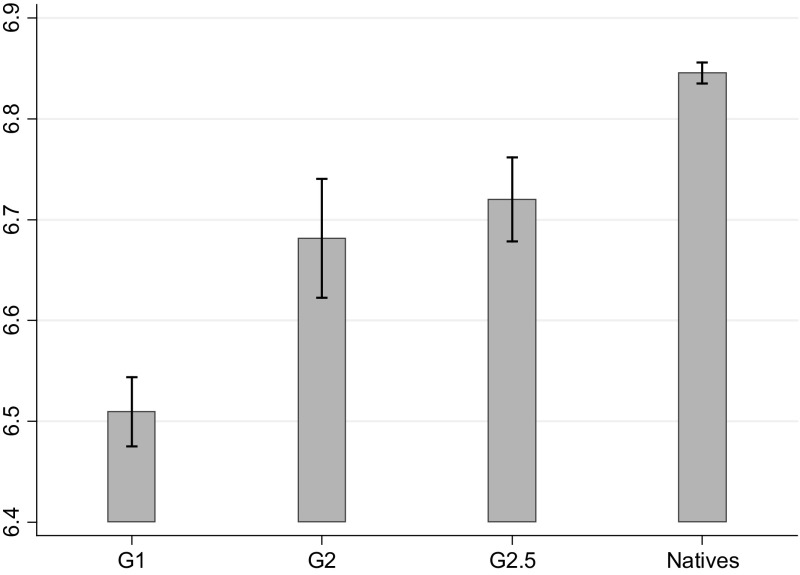
Adjusted mean life satisfaction scores by generation with 95% confidence intervals. *Note* Mean scores of life satisfaction are adjusted for wave, country of residence and continent of origin effects

In Fig. 1 the average scores on the life satisfaction variable for natives and migrants are presented. The scores presented here are adjusted for wave, country of residence and continent of origin to avoid confounding effects when comparing across immigrant generations. We find that people with a migration background show levels of life satisfaction that are, statistically speaking, significantly lower than those of natives. This confirms previous findings reported in the literature (Safi 2010). It is also evident that first generation immigrants report the lowest average level of life satisfaction. The gap between immigrants and natives reduces for later generations but remains statistically significant. At the same time differences in the average level of life satisfaction are not significantly different between the second and “2.5 generation”. The gaps in life satisfaction across the different immigrant generations and natives are small in magnitude.

### Multivariate Results

In Table 2 we present estimates from linear regression models predicting life satisfaction as a function of generation and social embeddedness controlling for the other covariates specified before. In this table, the coefficient of the dummy variables for the three immigrants groups (G1, G2 and G2.5) can be interpreted as the gap in life satisfaction between the corresponding groups and natives while adjusted for all control variables included in the model. In Model 1 (left panel of Table 2) only the control variables and generation are included. In line with the descriptive findings we see that life satisfaction of immigrants (G1) and that of persons with an immigrant origin (G2 and G2.5) is significantly lower than that of natives. As was shown in Fig. 1, the gap is biggest when comparing first generation immigrants to natives and is smallest for natives compared to second generations (without a difference between G2 and G2.5). Multivariate results confirm that the gaps in life satisfaction between immigrants and natives seem to be small (ranging from about 0.3–0.1 points, for first and “2.5” generation, respectively).

**Table 2:** Linear regression models predicting life satisfaction of immigrants and natives (N = 204,947)

Covariates	Models				
	(1)	(2)	(3)	(4)	(5)
Immigrant generation (ref: natives)					
G1	−0.29*** (0.02)	−0.26*** (0.02)	−0.24*** (0.02)	−0.26*** (0.02)	−0.22*** (0.02)
G2	−0.14*** (0.03)	−0.15*** (0.03)	−0.11*** (0.03)	−0.13*** (0.03)	−0.13*** (0.03)
G2.5	−0.10*** (0.02)	−0.12*** (0.02)	−0.09*** (0.02)	−0.10*** (0.02)	−0.10*** (0.02)
Female (ref: male)	−0.13*** (0.01)	−0.15*** (0.01)	−0.14*** (0.01)	−0.11*** (0.01)	−0.13*** (0.01)
Age (ref: 30–54)					
18–29	0.52*** (0.01)	0.42*** (0.01)	0.48*** (0.01)	0.49*** (0.01)	0.41*** (0.01)
55–65	0.12*** (0.01)	0.15*** (0.01)	0.11*** (0.01)	0.13*** (0.01)	0.14*** (0.01)
Type of area of residence (ref: rural)					
Big city	−0.10*** (0.01)	−0.10*** (0.01)	−0.11*** (0.01)	−0.10*** (0.01)	−0.11*** (0.01)
Small city	−0.12*** (0.01)	−0.12*** (0.01)	−0.12*** (0.01)	−0.12*** (0.01)	−0.12*** (0.01)
Education level (ref: upper secondary)					
Less than secondary	−0.30*** (0.02)	−0.27*** (0.02)	−0.23*** (0.02)	−0.25*** (0.02)	−0.20*** (0.02)
Lower secondary	−0.17*** (0.01)	−0.16*** (0.01)	−0.13*** (0.01)	−0.15*** (0.01)	−0.12*** (0.01)
Tertiary	0.31*** (0.01)	0.29*** (0.01)	0.27*** (0.01)	0.30*** (0.01)	0.25*** (0.01)
Activity status(ref. employed)					
In school	0.28*** (0.02)	0.19*** (0.02)	0.23*** (0.02)	0.26*** (0.02)	0.18*** (0.02)
Unemployed	−1.18*** (0.02)	−1.17*** (0.02)	−1.13*** (0.02)	−1.15*** (0.02)	−1.12*** (0.02)
Retired	−0.26*** (0.02)	−0.25*** (0.02)	−0.23*** (0.02)	−0.24*** (0.02)	−0.22*** (0.02)
Other	−0.37*** (0.01)	−0.37*** (0.01)	−0.31*** (0.01)	−0.35*** (0.01)	−0.31*** (0.01)
Partnership status(ref: in a partnership)					
Never married	−0.44*** (0.01)	−0.50*** (0.01)	−0.43*** (0.01)	−0.41*** (0.01)	−0.45*** (0.01)
Separated	−0.78*** (0.02)	−0.80*** (0.02)	−0.76*** (0.02)	−0.74*** (0.02)	−0.75*** (0.02)
Widowed	−0.83*** (0.03)	−0.84*** (0.03)	−0.79*** (0.03)	−0.75*** (0.03)	−0.75*** (0.03)
Child in home (ref: no)	0.02 (0.01)	0.04*** (0.01)	0.04*** (0.01)	0.01 (0.01)	0.04*** (0.01)
Meet		0.20*** (0.00)			0.14*** (0.00)
Activities			0.34*** (0.01)		0.25*** (0.01)
Intimate discussion (ref: no)				0.79*** (0.02)	0.57*** (0.02)
Constant	7.41*** (0.03)	6.49*** (0.03)	6.47*** (0.03)	6.59*** (0.03)	5.51*** (0.03)

* *p* < 0.10; ** *p* < 0.05; *** *p* < 0.01. All models include fixed effects for wave, country of residence and continent of origin

Models 2–4 stepwise introduce each of the three social embeddedness indicators. All of them are positively and significantly associated with life satisfaction: meeting people more often, a more intensive participation in social activities and having persons with whom one can discuss intimate matters are each associated with higher levels of life satisfaction.

In model 5, where the three indicators are included contemporaneously, the magnitude of the estimated coefficients is slightly reduced as a consequence of the positive correlation between the indicators (although not resulting in problems of collinearity; see “Table 4 in the Appendix”). However, the associations remain statistically significant at the 1% level. Social embeddedness seems to be crucial for life satisfaction and each of the different aspects captured by our three indicators seem to have an independent effect.

Comparing model 1 to the other models in Table 2, we observe that the initial gap in life satisfaction between first generation immigrants and natives is reduced when introducing the social embeddedness variables. The coefficients for G2 and G2.5, on the contrary, are not altered when the social embeddedness indicators are introduced. These findings are consistent with the descriptive statistics described above: compared to natives only first generation immigrants showed systematically lower average values on all three social embeddedness variables. So, part of the disadvantage of first generation immigrants in terms of life satisfaction can be attributed to the lower levels of social embededdness and thus lower levels of social support they can rely on.

The remaining gap in life satisfaction between natives and first generation immigrants (even when we account for social embeddedness) is small but not negligible from a substantive point of view. In fact, the estimated coefficient is similar in magnitude to some other independent variables in our model such as tertiary education or retirement status.

In the next step we analyse to what extent social embeddedness has the same effect across immigrant generations and natives. The results of these additional regression models where we tested this are shown in Table 3. Models 1–3 stepwise introduce interactions between immigrant generations and each of the three social embeddedness indicators. Model 4 presents the full model including all the variables. The two numerical indicators (“meet” and “activities”) where centred on the grand mean.^2^ In this case, the coefficient of the dummy variables for the three immigrant categories (G1, G2 and G2.5) can be interpreted as the gap in life satisfaction between the corresponding groups and natives for a hypothetical individual who report an average value for the social embeddedness variables interacted with the immigrant origin dummies. Interactions between the dummies for immigrant category dummies and centered social embeddednss variables measure the differential effect of the latter for each immigrant category as compared to natives. For brevity we do not show estimated coefficients for the covariates as they are similar to those reported in Table 2 (details available from the first author upon request).

**Table 3:** Linear regression models predicting life satisfaction of immigrants and natives allowing for interactions between generations and social embeddedness (N = 204,947)

Covariates	Models			
	(1)	(2)	(3)	(4)
Immigrant generation (ref: natives)				
G1	−0.26*** (0.02)	−0.24*** (0.02)	−0.29*** (0.05)	−0.27*** (0.05)
G2	−0.16*** (0.03)	−0.12*** (0.03)	−0.25** (0.10)	−0.26*** (0.10)
G2.5	−0.12*** (0.02)	−0.09*** (0.02)	−0.01 (0.08)	0.01 (0.08)
Meet	0.20*** (0.00)			0.13*** (0.00)
G1 × meet	0.00 (0.01)			0.01 (0.01)
G2 × meet	0.05*** (0.02)			0.07*** (0.02)
G2.5 × meet	0.02* (0.01)			0.03* (0.01)
Activities		0.35*** (0.01)		0.26*** (0.01)
G1 × activities		−0.04*** (0.02)		−0.05*** (0.02)
G2 × activities		−0.09*** (0.03)		−0.11*** (0.03)
G2.5 × activities		−0.00 (0.02)		−0.01 (0.02)
Intimate discussion			0.79*** (0.02)	0.57*** (0.02)
G1 × intimate discussion			0.04 (0.05)	0.05 (0.06)
G2 × intimate discussion			0.13 (0.10)	0.13 (0.10)
G2.5 × intimate discussion			−0.10 (0.08)	−0.12 (0.08)
Constant	7.49*** (0.02)	7.40*** (0.02)	6.59*** (0.03)	6.87*** (0.03)

* *p* < 0.10; ** *p* < 0.05; *** *p* < 0.01. All control variables (gender, age, type of area of residence, education, activity status, partnership status, presence of children in the home and fixed effects for wave, country of residence and continent of origin) are included. The variables “meet” and “activities” are centered on the grand mean

Some of the estimated coefficients of the interactions are statistically significant but never bigger in magnitude than that of the main effect meaning that the associations between social embeddedness and life satisfaction do not disappear for none of the different generation. Actually, for the first indicator, meeting with other people, the coefficients of interactions with G2 and G2.5 are statistically significant and positive meaning that this factor is even more important for both second-generation immigrant groups. On the contrary, participation in activities seems to be less strongly associated with life satisfaction for second generations compared to natives, but the association remains positive. Having persons to discuss intimate matters is equally important for all groups.

**Fig. 2:**
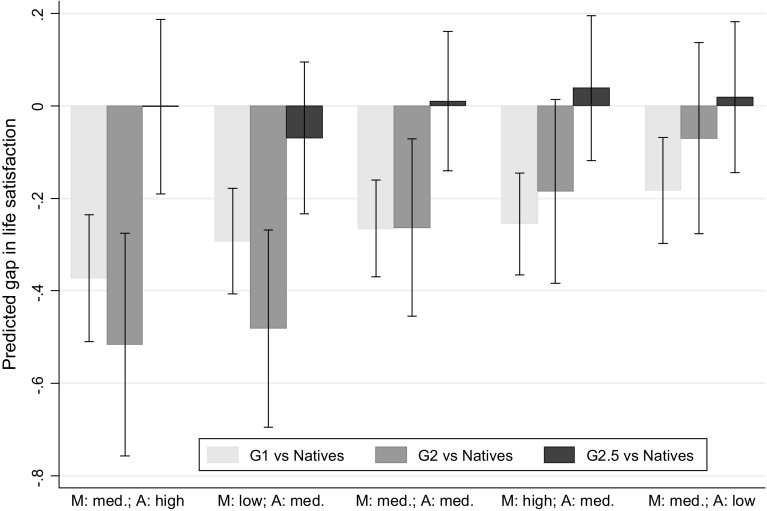
Predicted gap in life satisfaction of immigrants compared to natives with 95% confidence intervals. *Note* Predictions are based on the estimates of model 4 in Table 3 and obtained for some combinations of the values of the variables “meet” (M) and “activities” (A). Predictions are obtained fixing the value of the corresponding variable to the mean (med.), to a higher value (“high”) or a lower value (“low”). In particular, M: med., M: low and M: high correspond to the values: 4.9, 2, 6; A: med., A: low and A: high correspond to: 2.7, 1, 5. All the other covariates are held to the observed value and averaged out. Confidence intervals crossing the* horizontal zero-line* indicate a non significant gap in life satisfaction between an immigrant group and natives

These interaction effects imply interesting variability in the gap in life satisfaction among immigrants and natives that was partially hidden when studying the average gaps (estimated by model 5 in Table 2). Using the estimates of model 4 in Table 3, Fig. 2 illustrates the comparison between predicted life satisfaction of immigrants and natives for different combinations of “meet” and “activities”. Predictions are obtained fixing the value of the corresponding variable to the mean (“med.”), to a lower value (low”) or to a higher value (“high”). In particular, M: med., M: low and M: high correspond to the values 4.9 (the mean), 2 (corresponding to “Less than once a month”) and 6 (corresponding to “Several times a week”). On the other hand, A: med., A: low and A: high correspond to: 2.7 (the mean), 1 (“Much less than most”), 5 (“Much more than most”). For each person in the sample, all control variables are kept at the observed values and averaged out. The same is done for the variable “intimate” because the interactions with this variable and generations are not statistically significant.

Confidence intervals at the 95% level for testing the hypotheses that each gap is statistically different from zero are reported. Confidence intervals that cross the horizontal zero-line indicate a non-significant gap in life satisfaction between an immigrant group and natives (please note that comparing confidence intervals between them as such is not the key interest here and would result in an overestimation of the corresponding tests of significance).

We find that the estimated coefficient of the indicator for second generations (G2) in Model 4 of Table 3, that refers to an individual with average level of “meeting” and “social activities” and “without persons with whom discussing personal matters”, is bigger than in Model 5 of Table 2. The gap between second generations and natives reduces for people that meet often with friends and relatives (because the interactions between this variable and immigrants generation are positive) and increases for people that participate more in activities than other people (because its positive effect on life satisfaction is stronger for natives). This pattern is shown clearly in Fig. 2. Moving from the left to the right, gaps between G1 and natives and between G2 and natives are increasingly smaller. The gap between second generation immigrants and natives is statistically insignificant for people that meet with other people several times a week and among people that participate much more than most people in social activities. The gap between first generation immigrants and natives remains always statistically significant even though it changes by the different levels of social embeddedness. Finally, we notice that life satisfaction of 2.5 generation is never statistically different from that of natives. Actually, in the majority of the considered cases the point estimate is in favour of life satisfaction among the 2.5 generation.

### Further Analyses and Robustness Checks

Additionally to the main analyses reported above, we conducted a number of further analyses and robustness checks. First, we compared the findings we reported above using life satisfaction as dependent variable with those on happiness. As found in other studies, the reported levels of happiness are slightly higher, on average, than those for life satisfaction. However, the correlation between life satisfaction and happiness is very high and similar for natives (0.71) and across immigrants’ generations (the minimum was 0.68 for first generation immigrants and the maximum 0.70 for G2.5). Given these high correlations, it is not surprising that multivariate analyses using happiness as outcome variable showed very similar patterns with respect to those found for life satisfaction. We only notice that differences among immigrants and natives are somewhat smaller for happiness than they are for life satisfaction. On the contrary, the effects of the social embeddedness variables are similar for both happiness and life satisfaction.

Second, we run separate analyses by gender. Results for men and women are overall similar with one exception: the effect of the indicator for second generations is not statistically significant for women. Given the similarities in effects for men and women only the models on the pooled data are presented here (complete results for both analyses on happiness and those by gender are available from the first author upon request).

Finally, we used cross-validation and bootstrap (Efron and Gong 1983) to assess the stability of the interaction effects estimated in Table 3. As for cross-validation, we used k-fold as implemented by the Stata package *crossfold*. This procedure splits the data randomly into k partitions and for each partition it fits the model using the other k-1 groups only. We used the default number of folds (5). Since, the same models are estimated on different samples, to carry out the cross-validation analysis we had to use uncentered variables, meaning that estimated coefficients are not comparable to the original estimates in Table 3.^3^ However, here the interest is in assessing stability of models estimates. Therefore, in “Table 5 in the Appendix” we compare the 5 estimated models by the cross-validation procedure and a model estimated on the pooled sample but using uncentered variables. Table 5 reports the analyses for the complete model including all social embeddedness variables (as in the last column of Table 3) but the other analyses (available upon request) are similar. Results in Table 5 show that estimates are overall stable across the different samples both in terms of magnitude and standard errors. In some limited case significance change but this is expected as result of the different sample sizes.

As a second way of assessing stability of our results we used bootstrap. Bootstrap consists in drawing, with replacement, N observations from the N-observation original dataset. This process is replicated many times and each time the parameter(s) of interest is(are) estimated. We produced 500 replicates using the *bootstrap* package in Stata. This technique is typically employed to estimate standard errors in case it is difficult or impossible to obtain a variance estimator of the parameter of interest. It is also used as a robust approach to estimate standard errors. Similarly to cross-validation, this technique can be also used to assess stability of estimates. We used the approach for both goals focussing on the interactions between G2 and the embeddedness indicators in the final model of Table 3. We found that the bootstrap standard errors were very similar to those presented in Table 3. As for the stability of estimates, we calculated the average percent difference between the original estimates in Table 3 and each estimate obtained in the 500 replicates. Results indicate that estimates are stable. For example, results for the interactions with G2 are as follows: 1.1% (G2 × Meet), 2.7% (G2 × Activities) and 6.2% (G2 × Intimate discussion).

## Conclusions

In this paper we used data from the first 6 waves of the European Social Survey to study life satisfaction of immigrants and natives in 34 countries. Our study complements earlier work by taking a more comprehensive view on migrants’ life satisfaction across Europe using the most recent available data. Our study contributed to further knowledge comparing not only migrants and natives but by analysing the role and importance of social capital and embeddedness in social relations for life satisfaction of the individual.

Consistently with previous recent studies (Safi 2010; Kirmanoglu and Baslevent 2014), we found that life satisfaction of first generation migrants is lower than that of natives. At the same time our analyses show that here is a generational gradient with smaller gaps for the second and 2.5 generation children of immigrants.

Previous research has proposed different explanations for the lower life satisfaction of migrants compared to that of natives. For the best of our knowledge, we are the first to analyse the role of social embeddedness. Although effect sizes are overall not large, we did find that social embeddedness plays an important role in influencing migrants’ and natives’ life satisfaction. Descriptive results show that social embeddedness of first generation immigrants is lower than that of natives. On the contrary, there is no evidence that second generation migrants have lower levels of social embeddedness. Indeed, all the three measures of social embeddedness we considered are found to be positively related with life satisfaction and once they are controlled for the gap between first generation migrants and natives was substantially reduced. This suggests that part of the difference in life satisfaction between natives and migrants results from diverse levels of social embeddedness. This finding seems in line with studies that perceive migration as a disruptive effect for social relations in general. Whether more transnational ties can compensate for this disruption effect, cannot be assessed with our data. Also the role of close ethnic homogeneous or diverse networks cannot be studied with the data. Our findings nevertheless point to the precarious situation of migrants for whom relations after migration clearly need time to develop while at the same time being essential for their wellbeing.

Apart from the different *levels* of social embeddness among migrants and natives the type of relations also seems to matter. By including interaction terms between generations and different aspects of social embeddness indicators we showed heterogeneity in the relationship with life satisfaction between types of relationships. Previous studies have shown that social embeddness can be a protective factor for life satisfaction (Siedlecki et al. 2014). We found that this also holds for immigrants’ life satisfaction but the three measures we considered are not equally important for migrants and natives. While not having someone with whom to discuss personal matters is of the same importance for migrants and non-migrants, our results showed that meeting with other people is more important for migrants of the second and 2.5 generations than for natives. At the same time participation in social activities, tough important for both migrants and natives, had a weaker impact on migrants’ life satisfaction. Our findings show that it is important to take different measures of social relations and embeddedness into account. Future data collection efforts should potentially also explore the role of modern media to keep in touch with family and friends across borders and how this transnational embeddedness compares to the importance of having a local network.

All in all, our results point at important issues for policy in particular when it comes to disadvantages in social network for explaining life satisfaction among the children of immigrants. While most integration policies aim at first generation migrants our findings suggest that it is equally important to pay attention to the second generation. Young adults of immigrant origin may balance between their families and peers in the society they grow up in, resulting in potentially more conflictous family relations and a precarious situation in terms of their social embeddeddness. Stimulating social participation of immigrants and their children seems according to our findings an important way to strengthen social relations and helping wellbeing among these groups.

Although our study adds to knowledge for both scientists and practitioners, it also had limitations. First of all, with our cross sectional data we could only measure the social relations at one point in time. We pinpointed some differences between immigrant generations but it would be equally interesting to analyse the effects of duration of stay in the host country by first generation migrants Although Safi (2010) found that first generation migrants’ length of stay in the host country only marginally influences migrants’ life satisfaction it would be interesting to analyse the interrelationship over time. Especially for migrants with very different motives for migration; forced or voluntary migration, for example, could have potentially very different effects on a person’s wellbeing and life satisfaction. Unfortunately due to the cross-sectional nature of the ESS data these more dynamic aspects could not be studied. Moreover, information of migration motives is not available in the ESS. It would be an asset for future data collection efforts (as so far these data are absent) to take a longitudinal perspective and analyse the interrelation between migration motives, length of stay and changes in migrants’ life satisfaction.

Given the cross-sectional nature of the data we use, reverse causality between the variables studied cannot be ruled out. For example, not only meeting with other people may foster life satisfaction, but it could also be hypothesized that people with higher subjective wellbeing are more likely to meet with other people. Future studies using longitudinal data that cover people of immigrant and non-immigrant origin should address these issues further.

Another limitation of the data we used is that first generation immigrants that are included in the ESS are probably a selected group because only persons that speak one of the official languages of the country where the survey was conducted were interviewed. This means that among interviewed first generation immigrants those with higher education and those who stayed in the country longer are likely to be overrepresented. However, if this is the case the negative gap in social embeddeness and life satisfaction we found between first generation immigrants and natives, is probably even underestimated.

In this paper we focussed on comparisons between migrants and natives’ life satisfaction. An interesting avenue for future research is to analyse how country of origin and destination characteristics (such as linguistic and cultural distance) influence migrants’ life satisfaction. The extent to which also the size of the own community and levels of segregation are relevant in terms of social embeddedness are also aspects that could be covered in future studies. Nevertheless our study pointed out that also in times of increased mobility and easier communication options, the social relations a person has are still essential for individual life satisfaction. The fact that this is even more crucial for migrants calls for attention to facilitate their embeddedness in order to improve wellbeing. In light of the increasing share of people of immigrant origin across Europe this needs more attention by scholars and policy makers alike.

## Appendix

See Tables 4 and 5.

**Table 4:** Association among selected independent variables (N = 204,947)

	With partner	Never married	Separated	Widowed	Child in home	Meet	Activities
Child in home	0.62	−0.66	−0.07	−0.08			
Meet	−0.22	0.30	−0.05	−0.11	−0.16		
Activities	−0.04	0.09	−0.04	−0.09	−0.07	0.37	
Intimate discussion	0.10	0.05	−0.14	−0.26	0.06	0.27	0.27

The measure of association employed is: tetrachoric correlation for pairs of binary variables, polychoric correlation for pairs of numerical variables, and biserial correlation when one variable is binary and the other is numerical. All estimates are significant at the 1% level

**Table 5:** Cross-validation (fivefold) of the linear regression model predicting life satisfaction of immigrants and natives allowing for interactions between generations and all social embeddedness variables (corresponding to the last model in Table 3 in the paper) (N = 204,947)

Covariates	Samples					
	Original	Fold 1	Fold 2	Fold 3	Fold 4	Fold 5
Immigrant generation (ref: natives)						
G1	−0.18** (0.07)	−0.18** (0.08)	−0.12 (0.08)	−0.23*** (0.08)	−0.20** (0.08)	−0.18** (0.08)
G2	−0.33** (0.13)	−0.34** (0.14)	−0.32** (0.15)	−0.34** (0.15)	−0.33** (0.15)	−0.30** (0.15)
G2.5	−0.11 (0.10)	−0.09 (0.11)	−0.12 (0.11)	−0.18 (0.11)	−0.04 (0.11)	−0.12 (0.11)
Meet	0.13*** (0.00)	0.13*** (0.00)	0.13*** (0.00)	0.13*** (0.00)	0.13*** (0.00)	0.13*** (0.00)
G1 × meet	0.26*** (0.01)	0.26*** (0.01)	0.26*** (0.01)	0.26*** (0.01)	0.25*** (0.01)	0.26*** (0.01)
G2 × meet	0.57*** (0.02)	0.57*** (0.02)	0.58*** (0.02)	0.58*** (0.02)	0.58*** (0.02)	0.54*** (0.02)
G2.5 × meet	0.01 (0.01)	0.01 (0.01)	0.01 (0.01)	0.01 (0.01)	0.01 (0.01)	0.00 (0.01)
Activities	0.07*** (0.02)	0.08*** (0.02)	0.08*** (0.02)	0.08*** (0.02)	0.07*** (0.02)	0.07*** (0.02)
G1 × activities	0.03* (0.01)	0.02 (0.02)	0.03** (0.02)	0.01 (0.02)	0.03* (0.02)	0.04** (0.02)
G2 × activities	−0.05*** (0.02)	−0.05*** (0.02)	−0.06*** (0.02)	−0.04** (0.02)	−0.03 (0.02)	−0.06*** (0.02)
G2.5 × activities	−0.11*** (0.03)	−0.11*** (0.03)	−0.12*** (0.03)	−0.10*** (0.03)	−0.11*** (0.03)	−0.11*** (0.03)
Intimate discussion	−0.01 (0.02)	−0.01 (0.03)	0.01 (0.03)	0.01 (0.03)	−0.00 (0.03)	−0.03 (0.03)
G1 × intimate discussion	0.05 (0.06)	0.06 (0.06)	0.02 (0.06)	0.06 (0.06)	0.02 (0.06)	0.10 (0.06)
G2 × intimate discussion	0.13 (0.10)	0.13 (0.11)	0.14 (0.11)	0.08 (0.11)	0.18 (0.11)	0.13 (0.12)
G2.5 × intimate discussion	−0.12 (0.08)	−0.11 (0.09)	−0.19** (0.09)	−0.04 (0.09)	−0.19** (0.09)	−0.09 (0.09)
Constant	5.51*** (0.04)	5.53*** (0.04)	5.50*** (0.04)	5.51*** (0.04)	5.48*** (0.04)	5.56*** (0.04)
N	204,947	163,957	163,958	163,957	163,958	163,958

* *p* < 0.10; ** *p* < 0.05; *** *p* < 0.01. All control variables (gender, age, type of area of residence, education, activity status, partnership status, presence of children in the home and fixed effects for wave, country of residence and continent of origin) are included. Please, notice that differently from models in Table 3 in the paper variables “meet” and “activities” are not centered

## Footnotes

1 Data and documentation can be freely downloaded from http://www.europeansocialsurvey.org.

2 Centering a numerical variable is a common practice when this variable is interacted with another independent variable. In linear models, as ours, centering a variable does not alter the model from a statistical point of view. The statistical fit is the same and also the predicted values at each value of the interacted values are not altered (for further details see e.g., Afshartous and Preston 2011).

3 As already mentioned in note 2 at page 15, centering a variable only implies a reparametrization of the model that remains otherwise equivalent from a statistical point of view. In other words, it is only a different way of presenting the same results.
